# High-Risk Outcomes in In Vitro Fertilization Pregnancies for Women of a Very Advanced Maternal Age: Insights from a Multi-Hospital Study in Greece

**DOI:** 10.3390/jcm14041323

**Published:** 2025-02-17

**Authors:** Themistoklis Loukopoulos, Athanasios Zikopoulos, Efstratios Kolibianakis, Anastasia Vatopoulou, Fani Gkrozou, Sotirios Sotiriou, Athanasios Zachariou, Charikleia Skentou

**Affiliations:** 1 Department of Obstetrics and Gynecology, Medical School of Ioannina, University General Hospital, 45110 Ioannina, Greece; 2 Obstetrics and Gynecology, Royal Devon and Exeter Hospital Barrack Rd, Exeter EX 25 DW, UK; 3 3rd Department of Obstetrics and Gynecology, Aristotle University of Thessaloniki, 54128 Thessaloniki, Greece; 4 Department of Embryology, Faculty of Medicine, University of Thessaly, 38221 Larissa, Greece; 5 Department of Urology, Medical School of Ioannina, University General Hospital, 45110 Ioannina, Greece; zahariou@otenet.gr

**Keywords:** assisted reproduction, in vitro fertilization, aging, preeclampsia, gestational diabetes, preterm labor, low birth weight, placenta abnormalities

## Abstract

**Background:** In vitro fertilization (IVF) has transformed infertility treatment, yet it is associated with increased risks of adverse perinatal outcomes, particularly in women of advanced maternal age. This study aimed to investigate the prevalence of complications such as preeclampsia (PE), gestational diabetes mellitus (GDM), preterm labor (PTL), low birth weight (LBW), and placental abnormalities (PA) among women over 50 undergoing assisted reproductive technology (ART) in Greece, where the eligibility age limit has been recently raised to 54 years. **Methods:** We conducted a retrospective analysis of pregnancy outcomes in women over 50 compared to those under 50, utilizing medical records mainly from University Hospital of Ioannina but also from other public hospitals and private clinics in Greece. **Results:** Our findings indicate that women over 50 face an increased risk of developing preeclampsia (PE) by 4.61 times, GDM by 1.69 times, PTL by 1.82 times, LBW by 1.67 times, and PA by 3.92 times. **Conclusions:** These results underscore the need for heightened awareness and the monitoring of pregnancy complications in this demographic, informing clinical strategies to improve maternal and neonatal outcomes.

## 1. Introduction

The development of in vitro fertilization (IVF) has revolutionized the management of infertility, offering an effective therapeutic option for individuals across diverse age groups. Greece attracts over 1000 women annually, owing to its notable IVF success rates, progressive legislative framework, and access to advanced medical infrastructure equipped with cutting-edge technologies. These factors have positioned Greece as a prominent hub for reproductive tourism, facilitating access to assisted reproductive technologies (ART) for international patients [1].

The recent expansion of age restrictions for IVF eligibility in Greece has led to an increase in the number of women over 50 seeking treatment. Similar trends are being observed globally, driven by socioeconomic factors that contribute to an increasing desire among women to conceive at an advanced maternal age. However, advanced maternal age (AMA) remains consistently associated with an elevated risk of adverse pregnancy outcomes. It is important to note that the significantly higher reliance on egg donation among women of advanced maternal age (AMA) distinguishes these pregnancies from those of younger women. This distinction complicates the assessment of age-related risks, as adverse outcomes in older women undergoing IVF may result not only from advanced maternal age itself but also from the specific consequences of utilizing donor eggs.

Complications frequently associated with pregnancies in AMA women include placental abnormalities, low birth weight, preterm labor, preeclampsia, and gestational diabetes mellitus (GDM). Recent studies have provided further insights into these risks. For example, Sugai et al. (2023) conducted a systematic review and meta-analysis focusing on pregnancies in women over 45 years of age, reporting a significantly higher incidence of adverse outcomes such as preeclampsia and GDM [2]. Similarly, Thilaganathan investigated the cardiovascular-placental axis in the context of preeclampsia, identifying mechanisms that impair physiological adaptations during pregnancy in older mothers [3].

In addition, Sacha et al. evaluated live births from both fresh and frozen embryo transfers, emphasizing the critical influence of placental health on IVF pregnancy outcomes [4]. Their findings indicate that pregnancies following frozen embryo transfers exhibit more anatomical and vascular placental pathology compared to those from fresh transfers, despite similar maternal outcomes. Furthermore, Masoudian et al. (2023) identified a concerning correlation between oocyte donation pregnancies and the heightened incidence of preeclampsia and gestational hypertension, underscoring the unique risks associated with donor egg pregnancies [5].

This retrospective study aimed to compare the prevalence of pregnancy complications—specifically preterm labor (PTL) [6], low birth weight (LBW), placental abnormalities (PA), gestational diabetes mellitus (GDM), and preeclampsia (PE)—among women over 50 undergoing IVF in Greece with those under 50 [7]. By identifying these disparities, we seek to enhance awareness of the risks associated with advanced maternal age (AMA) in assisted reproductive technology (ART) pregnancies and to inform clinical strategies aimed at improving maternal and neonatal outcomes.

## 2. Materials and Methods

This retrospective cohort study was conducted at the University Hospital of Ioannina and other public hospitals and private clinics across Greece from 2014 to 2024.

### 2.1. Participant Selection

Women who underwent successful in vitro fertilization (IVF) procedures and delivered at the University Hospital of Ioannina during the study period were identified through computerized hospital discharge records. Additionally, data were collected from women over 50 years of age who received IVF treatment at other public hospitals and private clinics throughout Greece. A total of 383 participants were selected based on the following inclusion criteria: women who had undergone a standardized IVF procedure that included both oocyte donation and autologous cycles. Specifically, women over the age of 50 were more likely to receive donor oocytes due to their menopausal status, as they had not undergone ovarian stimulation.

### 2.2. IVF Procedures

Women who underwent ovarian stimulation received a gonadotropin-based protocol, with dosages tailored to individual ovarian responses. Ovarian response was monitored through regular ultrasound examinations and serum hormone level assessments (estradiol, progesterone, and LH), allowing for adjustments in medication as necessary. Oocyte retrieval was performed using transvaginal ultrasound-guided aspiration.

Endometrial preparation was conducted for both frozen and donor cycles, which included the administration of estrogen and progesterone to promote endometrial development necessary for successful implantation.

Of the 383 participants, 296 women were categorized as the “Below 50 Years” group, while 87 women were designated as the “Above 50 Years” group. In the “Below 50 Years” group, 73 women (24.7%) utilized donor eggs, and 156 women (52.7%) underwent a stimulation protocol; 177 women (59.7%) received endometrial preparation. In contrast, the “Above 50 Years” group had 81 women (93.1%) using donor eggs, and all received endometrial preparation.

### 2.3. Outcomes and Data Collection

A retrospective analysis was performed to compare the incidence of pre-eclampsia, gestational diabetes mellitus, preterm labor, low birth weight, and placental abnormalities (including placenta previa, placenta accreta, and placental abruption) between the two groups. Twin pregnancies were documented, with 107 women (36%) in the “Below 50 Years” group experiencing twin pregnancies, although two cases resulted in singleton deliveries due to fetal demise at 14 and 21 weeks of gestation, respectively. In the “Above 50 Years” group, twin pregnancies were observed in 38 out of 87 women (43.6%).

### 2.4. Prenatal Care and Delivery

Prenatal care and ultrasound examinations were conducted by physicians certified by the Fetal Medicine Foundation (FMF). All deliveries were performed in tertiary care hospitals exclusively via cesarean section. Women identified as being at moderate or high risk for preeclampsia were prescribed 150 mg of aspirin daily, in accordance with the latest guidelines from the European Society of Hypertension.

## 3. Statistical Analysis

All categorical outcomes, along with the age group classifications, were summarized using frequencies and percentages. The statistical relationships between each outcome and the age groups were evaluated using Pearson’s Chi-Square test, following verification of its underlying assumptions. Odds ratios (ORs) with corresponding 95% confidence intervals (CIs) were calculated to quantify the strength of these associations. Data analysis was performed using SPSS software (version 26.0), with a significance threshold set at *p* < 0.05 for all tests.

## 4. Results

The sample comprised 383 women, 22.7% of whom were aged between 50 and 54 years (above 50 years group). Recorded adverse outcomes included preeclampsia, gestational diabetes, preterm labor, low birth weight, and placenta abnormalities. The analysis revealed statistically significant differences between age groups for all examined adverse outcomes, except for a low birth weight.

The adverse outcomes in two groups are displayed in Table 1.

The odds of preeclampsia were 4.61 times higher in the above 50 years group compared to the below 50 years group (95% CI: 2.31–9.19; *p* < 0.001). Similarly, the odds of GDM were 1.69 times higher in the above 50 years group (95% CI: 1.01–2.82; *p* = 0.043).

For preterm labor, the odds were 1.82 times higher in the above 50 years group (95% CI: 1.01–3.27; *p* = 0.043). The odds regarding low birth weight were 1.67 but not significantly different to 1 with a 95% CI: (0.90–3.09; *p* = 0.101). Lastly, the odds of placenta abnormalities were 3.92 times higher (95% CI: 2.22–6.91; *p* < 0.001).

**Figure 1:**
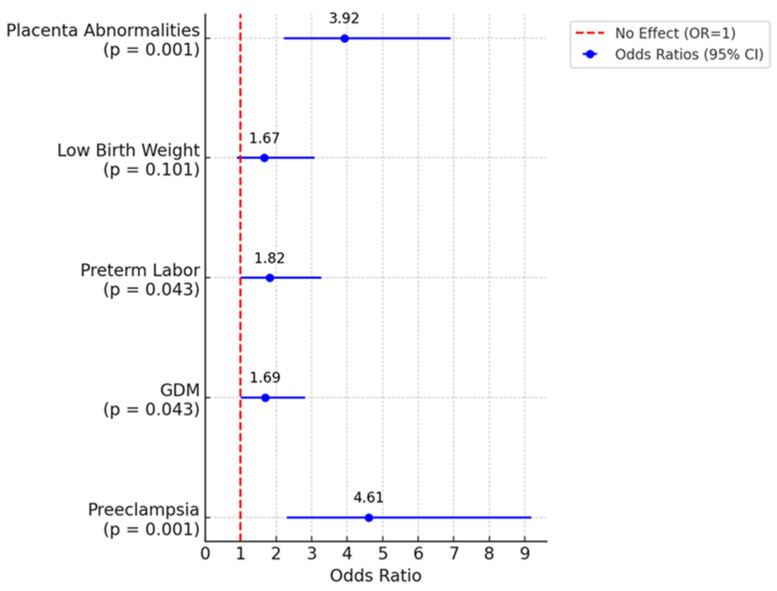
Forest plot comparing pregnancy complications between women aged above 50-year-old and those below 50-year-old.

These statistical differences are illustrated in Figure 1.

## 5. Discussion

This retrospective cohort study aimed to evaluate the association between maternal age and the incidence of common pregnancy complications in pregnancies achieved through in vitro fertilization (IVF). The findings demonstrated a significant increase in the prevalence of major complications among women aged over 50 years compared to those under 50 years. These complications included preeclampsia, gestational diabetes mellitus, preterm labor, and placental abnormalities.

### 5.1. Preeclampsia

The existing literature indicates that pregnancies conceived via in vitro fertilization (IVF) have a higher risk of developing preeclampsia (PE) compared to spontaneous pregnancies [8,9,10,11]. In the present study, the incidence of preeclampsia among women under 50 years of age (6.1%) aligns with previous findings. However, the incidence observed in women over 50 years of age (23%) was markedly higher than the rates reported in earlier studies.

The exact etiology of preeclampsia (PE) remains incompletely understood; however, improper placentation is widely recognized as a critical factor in its pathogenesis [11,12,13]. This process involves deficient trophoblast invasion, inadequate placental implantation, insufficient spiral artery remodeling, and placental hypoperfusion [14,15]. Additional factors potentially contributing to PE include immunological dysfunction, inflammation, and genetic predispositions. The role of in vitro fertilization (IVF) and associated risk factors in the development of PE remains a subject of debate.

A retrospective population analysis identified multiple pregnancies as a primary contributor to the increased prevalence of PE in assisted reproductive technologies (ARTs) [16]. This finding may partially explain the significant differences observed in our study, where twin pregnancies were more frequent among older women (43.6%) compared to younger women (36%). Another interesting finding in our study concerns endometrial preparation. While many women under 50 years old received stimulation, not all of them received endometrial preparation. In contrast, every woman in the “Above 50 Years” group received endometrial preparation as a part of their treatment, which is essential due to their menopausal status. This difference in estrogen and progesterone administration could help explain why we observed a higher incidence of preeclampsia (PE) in the older group.

While prior studies have reported an elevated risk of PE in overweight or obese women, no significant differences in body mass index (BMI) were identified between the age groups in our study [17,18].

Despite the multifactorial etiology of preeclampsia (PE), poor trophoblastic invasion and improper placentation—often linked to endometrial dysplasia during ovarian stimulation therapy—are considered key triggers for its onset [16]. In IVF pregnancies, the embryo is transferred to the uterine cavity via the cervix, and the hormonal alterations associated with IVF can disrupt the establishment of the maternal–fetal interface, potentially resulting in defective placental function [9].

A recent systematic review and meta-analysis identified advanced maternal age as an independent risk factor for PE, along with other complications such as gestational diabetes, stillbirth, intrauterine growth restriction, and placenta previa [19]. These complications may be associated with poor placentation and the progressive decline in uterine vascular endothelial function that occurs with aging [20,21,22]. The findings of our retrospective study align with these observations, further substantiating the evidence on the relationship between advanced maternal age, IVF, and adverse pregnancy outcomes.

### 5.2. Gestational Diabetes Mellitus

Gestational diabetes mellitus (GDM) is the most prevalent metabolic disorder during pregnancy, characterized by insulin resistance and an insufficient compensatory function of pancreatic islet β cells [23]. The higher incidence of GDM in women of advanced maternal age is thought to be linked to age-related dysregulation of lipid metabolism and increased oxidative stress [24]. Emerging evidence suggests that GDM has a multifactorial etiology, influenced by both genetic and environmental factors. Notably, interactions between maternal age, body mass index (BMI), and ethnicity play a significant role in the development of GDM [20].

Assisted reproductive technology (ART) is associated with an increased risk of gestational diabetes mellitus (GDM), with studies reporting a 1.59-fold higher risk in ART-conceived pregnancies compared to spontaneously conceived pregnancies [25]. This risk is particularly pronounced in women undergoing in vitro fertilization (IVF) compared to those utilizing intracytoplasmic sperm injection (ICSI) [23]. Additionally, maternal age has been shown to have a significant positive correlation with the risk of GDM, with a 7.90% increase in risk for each additional year of maternal age [26,27]. Our findings are consistent with the existing literature, demonstrating a higher incidence of GDM in older women (24.7% in the younger group vs. 35.6% in women over 50 years old).

### 5.3. Preterm Labor

The World Health Organization (WHO) defines preterm labor (PTL) as any birth occurring before 37 weeks (259 days) of gestation, irrespective of whether the pregnancy involves a single or multiple gestation [28]. PTL is a leading contributor to increased fetal and neonatal mortality and morbidity, even in high-income countries [29]. While the precise etiology of PTL remains unknown, it is believed to result from either early idiopathic activation or pathological deviations from the processes of normal labor.

Current understanding suggests that PTL is a multifactorial condition initiated by various triggers, including infection or inflammation, uterine–placental ischemia or hemorrhage, uterine overdistension, stress, and immune-mediated mechanisms [30].

The association between preterm labor (PTL) and advanced maternal age (AMA) remains a subject of ongoing investigation [31]. Multiple studies have reported a link between AMA and an elevated risk of PTL, even after adjusting for confounding factors such as body mass index (BMI), education level, parity, pre-existing health conditions, and the method of conception [31]. In our study, women of very advanced maternal age exhibited a 1.82-fold increased likelihood of experiencing PTL compared to younger women. Although this association did not reach statistical significance, it indicates a potential trend consistent with the existing literature.

Interestingly, the older cohort in our study also showed higher rates of preeclampsia and gestational diabetes mellitus, both of which are well-documented risk factors for PTL [31,32,33]. Therefore, the observed increased risk of PTL in this group may be partially attributable to these underlying complications.

### 5.4. Low Birth Weight

Low birth weight (LBW) is defined by the World Health Organization (WHO) as a birth weight of less than 2500 g (up to and including 2499 g), irrespective of gestational age. It is further categorized into very low birth weight (VLBW) for infants weighing less than 1500 g and extremely low birth weight (ELBW) for those under 1000 g [34]. LBW is typically associated with preterm delivery, intrauterine growth restriction (IUGR), or a combination of both factors [35]. In contrast, “small for gestational age” (SGA) specifically refers to newborns with a birth weight below the 10th percentile for their gestational age [34]. This study focused exclusively on infants with birth weights below 2500 g.

The literature suggests that LBW risk parallels the risk of preterm labor (PTL), with both increasing with maternal age [36]. In our analysis, women over 50 years of age demonstrated a 1.67-fold higher likelihood of delivering LBW infants compared to younger women. However, this association did not reach statistical significance. The observed elevated risk of LBW is likely attributable to the higher incidence of preterm labor among older women in our study.

A well-established link exists between fetal growth restriction (FGR) and placental trophoblast aging [37]. Placentas affected by FGR often exhibit inadequate vascularization, reduced vascular branching, and diminished capillary density. Additionally, these placentas display increased rates of apoptosis, decreased cellular proliferation, and an elevated presence of syncytial nuclear aggregates (SNAs) [38]. While physiological placental aging is a normal process, abnormal or premature placental senescence can disrupt key functions, particularly nutrient and oxygen exchange, thereby contributing to adverse pregnancy outcomes [39].

Recent research indicates that advanced maternal age is associated with reduced expression of the anti-aging protein α-Klotho in placental trophoblasts. This reduction accelerates premature senescence and diminishes placental functionality, potentially leading to placental malformations and poor perinatal outcomes, such as FGR and low birth weight (LBW) [40].

### 5.5. Placental Abnormalities

Advanced maternal age has been strongly associated with compromised placental function, often attributed to reduced uterine blood flow, uteroplacental hypoperfusion, and increased prevalence of extensive placental infarctions. These factors contribute to a heightened risk of hemorrhagic conditions and placental abnormalities, which are more common in older women [41].

In our study, we investigated three categories of placental abnormalities: placenta previa, placenta accreta, and placental abruption [42]. According to the existing literature, the risks of these complications are elevated in pregnancies conceived through in vitro fertilization (IVF) compared to spontaneous conceptions, with reported prevalence rates of 4.8% for placenta previa, 4.7% for placenta accreta, and 1% for placental abruption. The absolute risk of these complications further increases with maternal age [42].

The results of our cohort were consistent among women under 50 years old, since the incidence of combined placental abnormalities in this group was 11.8%. However, this rate rose markedly with age, reaching 34.5% in women over 50. This significant difference may be attributed to the higher use of donor eggs, which occurred in 24.7% of the younger group and 93.1% of the older group. IVF using donor eggs is associated with a greater risk of placental anomalies, likely due to a combination of immunological, vascular, and endometrial factors.

In donor egg pregnancies, the genetic disparity between the mother and fetus induces heightened immunological activity at the maternal–fetal interface, resembling a host-versus-graft rejection. This response is characterized by severe chronic deciduitis, extensive fibrinoid deposition, and enhanced villitis of unknown origin, all of which impair placental development and functionality [43]. Furthermore, the absence of a corpus luteum in most donor egg pregnancies above 50 years of age reduces endometrial receptivity and decidualization, creating a suboptimal environment for trophoblast invasion and vascular remodeling.

Genome-wide studies have also identified altered expression of coagulation and immunological regulatory proteins in donor egg pregnancies, which contribute to placental dysfunction and complications such as preeclampsia and fetal growth restriction [44]. These mechanisms likely explain the increased risk of placental abnormalities observed in our study, particularly among the older age group undergoing IVF with donor eggs.

### 5.6. Impact of Egg Donation on Outcomes Between Two Groups

Pregnancies resulting from egg donation tend to have a higher chance of developing complications—such as preeclampsia and the rest of the conditions we included in our study—as compared to spontaneous conception and in vitro fertilization (IVF) with autologous eggs.

Keukens’ most current and extensive meta-analysis, published in 2022, evaluated the risk of developing preeclampsia and severe preeclampsia in pregnancies resulting from spontaneous conception, IVF with autologous eggs, or egg donation. Specifically, among the group that received egg donation and those who achieved pregnancy naturally, the likelihood of preeclampsia was approximately five times greater in the former group (OR: 5.09, 95% CI: 4.29–6.04), while the risk for severe preeclampsia was over seven times greater in the first group (OR: 7.42, 95% CI: 4.64–11.88). The increased risk associated with egg donation remained significant when compared to the IVF group using autologous eggs. In this case, the risk of both preeclampsia and severe preeclampsia was approximately three times higher in pregnancies with egg donation (OR: 2.97, 95% CI: 2.49–3.53, OR: 2.97, 95% CI: 2.15–4.11, respectively) [45].

Regarding the rates of egg donor usage in our retrospective study, among women under 50, it was 24.7%, while in participants over 50 years old, it was 93.1%. Statistical analysis showed that the likelihood of preeclampsia was 4.6 times higher in the latter group (6.10% vs. 23.00%, OR: 4.61 (2.31–9.19) *p* < 0.001). Considering the aforementioned literature, the significant difference observed in the rates of egg donation between the two groups may explain part of the markedly increased frequency of preeclampsia in the older age group of our study.

Other placental-related conditions, including placenta previa, placenta accreta, and placenta abruption, may occur more frequently in pregnancies resulting from oocyte donation. This higher risk is likely due to a variety of factors, including aberrant immunological responses at the fetal–maternal interface, abnormalities in endometrial receptivity, and underlying maternal features. These variables may contribute to placental dysfunction, resulting in a greater frequency of complications that impact placentation and overall pregnancy outcomes in this patient population [44].

Besides preeclampsia, pregnancies achieved through oocyte donation tend to have a higher chance of complications, such as preterm birth (PTB) and low birth weight (LBW), too. A systematic review and meta-analysis by Mascarenhas showed an increased risk of PTB following fresh embryo transfer in OD pregnancies compared to AO IVF pregnancies (OR 1.45, 95% CI 1.20–1.77). Similarly, the risk of LBW is higher after fresh embryo transfer in OD pregnancies than in AO IVF pregnancies (OR 1.34, 95% CI 1.12–1.60). When examining preterm birth before 32 weeks and very low birth weight, the likelihood of both outcomes remained higher in the OD group (OR 2.14, 95% CI 1.40–3.25 for early PTB and OR 1.51, 95% CI 1.17–1.95 for very low birth weight, respectively) [46].

Concerning gestational diabetes mellitus, there is a disagreement in the literature about whether it is independently influenced by oocyte donation. A meta-analysis by Moreno–Sepulveda found a significantly increased risk of GDM in singleton pregnancies resulting from oocyte donation compared to those from IVF, with an odds ratio of 1.27 (95% CI: 1.0, 1.56) [47]. In contrast, Storgaard’s meta-analysis reported a non-significant pooled risk estimate (aOR: 1.33, 95 %CI: 0.71, 2.50) [48].

It is important to acknowledge the inclusion of a limited number of participants, as the age limit for in vitro fertilization (IVF) in Greece was only recently raised to 54 years. This constraint highlights the need for cautious interpretation of our findings. Nonetheless, despite this limitation, statistical analyses revealed significant differences across all examined outcomes, with the exception of low birth weight (LBW). Although the difference in low birth weight (LBW) was not statistically significant, the direction and magnitude of the observed difference were consistent with the trends seen in other adverse outcomes among older vs. younger women. This finding may reflect a lack of statistical power as a potential explanation for the absence of significance. Alternatively, other factors, such as the higher utilization of frozen embryos in this age group compared to younger women, may also contribute to this result. To enhance the applicability and robustness of our findings, future research should aim to include larger and more diverse samples of women over the age of 50.

Moreover, while the study accounted for BMI as a potential confounder, other lifestyle factors and pre-existing health conditions were not included due to data limitations. Factors such as smoking, physical activity, and socio-economic status are known to influence pregnancy outcomes and could interact with advanced maternal age (AMA) to amplify risks. Additionally, the prevalence of pre-existing conditions like hypertension or diabetes, which are more common in AMA populations, may have contributed to the observed complications. Future studies should integrate detailed data on these variables to enhance the understanding of their independent and combined effects on maternal and neonatal outcomes in this high-risk group.

Healthcare providers should engage in comprehensive pre-treatment counseling that addresses the unique risks and benefits associated with IVF in women over 50. This includes discussing potential complications, such as all age-related risks mentioned in this paper. It is essential to develop individualized treatment plans that take into account the patient’s overall health and reproductive goals.

## 6. Conclusions

Our retrospective study provides valuable insights into the relationship between very advanced maternal age and the increased incidence of pregnancy complications following in vitro fertilization (IVF) therapy in Greece. The findings underscore the significantly higher risks faced by women over 50 years of age compared to their younger counterparts. Women in this age group were found to have a markedly increased risk of preeclampsia, gestational diabetes, preterm labor, and placental abnormalities. These results contribute to the growing body of literature exploring the complex interplay between advanced maternal age and adverse perinatal outcomes. They emphasize the importance of providing comprehensive prenatal care and informed decision-making for older women pursuing IVF therapy. Consequently, clinicians should consider the high-risk profile of women of advanced maternal age and provide appropriate counseling and close monitoring for this population. To build upon these findings, further research with larger sample sizes is essential to confirm the observed associations and investigate the underlying mechanisms driving these relationships. Such research will ultimately inform clinical practice and support interventions aimed at improving maternal and neonatal health outcomes in this high-risk group.

## Figures and Tables

**Table 1:** Adverse outcomes in the two age groups.

	Age					
	Below 50 Years		Above 50 Years			
	*n* = 296	%	*n* = 97	%	OR (95% CI)	*p*
Preeclampsia	18	6.10%	20	23.00%	4.61 (2.31–9.19))	<0.001
Gestational diabetes	73	24.70%	31	35.60%	1.69 (1.01–2.82)	0.043
Preterm labor	44	14.90%	21	24.10%	1.82 (1.01–3.27)	0.043
Low birth weight	40	13.50%	18	20.70%	1.67 (0.90–3.09)	0.101
Placenta abnormalities	35	11.80%	30	34.50%	3.92 (2.22–6.91)	<0.001

## Data Availability

The data presented in this study are available on request from the corresponding author.
